# Recurrence of thymic neuroendocrine carcinoma 24 years after total excision: A case report

**DOI:** 10.3892/ol.2013.1327

**Published:** 2013-05-01

**Authors:** GOUJI TOYOKAWA, KENICHI TAGUCHI, MIYAKO KOJO, RYO TOYOZAWA, EIKO INAMASU, YOSUKE MORODOMI, YOSHIMASA SHIRAISHI, TOMOYOSHI TAKENAKA, FUMIHIKO HIRAI, MASAFUMI YAMAGUCHI, TAKASHI SETO, MITSUHIRO TAKENOYAMA, YUKITO ICHINOSE

**Affiliations:** 1 Department of Thoracic Oncology, Institute for Clinical Research, National Kyushu Cancer Center, Fukuoka 811-1395, Japan; 2 Cancer Pathology Laboratory, Institute for Clinical Research, National Kyushu Cancer Center, Fukuoka 811-1395, Japan

**Keywords:** thymic neuroendocrine carcinoma, recurrence, long-term survival, surgery

## Abstract

A 77-year-old male presented with chest pain in March 2012. The individual had undergone surgery for an anterior mediastinal tumor 24 years earlier and the pathological diagnosis was that of a thymoma. The patient underwent a medical check-up every 6 months for the next 20 years. However, ∼3 years following the final check-up, sudden chest pain was reported and the patient was referred again. Computed axial tomography revealed a mediastinal mass adjacent to the left lung, pericardium and sternum. There was no apparent invasion to the adjacent structures. The patient underwent surgical resection following a diagnosis of recurrent thymoma. A posterolateral thoracotomy was performed under video-assisted thoracoscopy. Severe adhesions were observed around the tumor, which appeared to invade the left lung and pericardium, but not the chest wall. The tumor was extirpated in combination with partial resection of the left lung and pericardium. The pathological diagnosis of the tumor was of a well-differentiated neuroendocrine carcinoma (NEC) of the thymus. The specimen that was excised 24 years earlier was re-examined by a pathologist and was reported to exhibit the same histology. Primary NECs of the thymus are rare among anterior mediastinal tumors and the 5-year survival rate is ∼30%. The present case study reports a case of a thymic NEC and describes the pathological and clinical features.

## Introduction

Thymic neuroendocrine carcinomas (NECs) are rare and have been estimated to account for 2–4% of all anterior mediastinal tumors (1). Local and distal metastases frequently develop following surgical excision of these tumors (2–4). A previous study by Fukai *et al* showed that recurrence occurred 4–99 months after surgery (3) and recurrence after as long as 9 years has been described (5,6). However, to the best of our knowledge, no studies have discussed the development of recurrence >20 years after total excision. Although the optimal therapeutic modality for the treatment of recurrent disease has not been determined, more aggressive treatment, including re-excision of recurrent tumors, may be required to reduce the incidence of local recurrence and distant metastasis and to improve survival. The current study presents a rare case of a recurrent neuroendocrine tumor in the thymus developing 24 years after total excision. Written informed consent was obtained from the patient.

## Case report

### Clinical presentation

**Figure 1.**
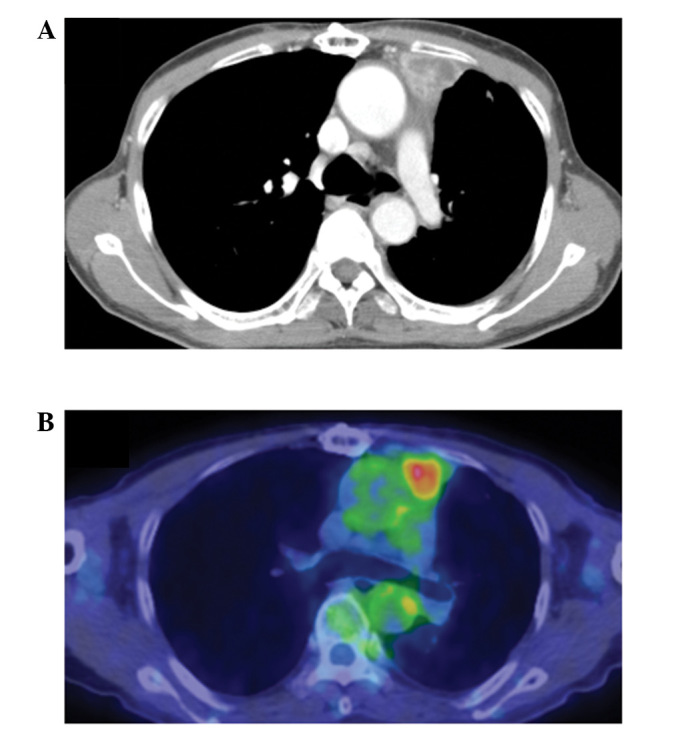
Imaging observations. (A) CT revealing an anterior mediastinal tumor with a maximum size of ∼3 cm. (B) PET/CT scan revealing abnormal uptake of ^18^F-fluorodeoxyglucose in the tumor. CT, computed tomography; PET, positron emission tomography.

A 77-year-old male was referred for an evaluation of an acute onset of chest pain. The patient had undergone a thymectomy via a median sternotomy for an anterior mediastinal tumor 24 years previously. The pathological diagnosis was of a World Health Organization (WHO) type B3 thymoma classified as pathological stage I due to the absence of capsular invasion (Masaoka classification). Regular medical check-ups had been performed twice a year for 20 years after the surgery and had been completed without evidence of recurrence. However, ∼3 years after the final check-up, a sudden onset of left-sided chest pain was reported and the patient was referred again. Laboratory examinations revealed elevated C-reactive protein levels (3.53 mg/dl), but no other abnormal levels of any tumor markers, including neuron specific enolase. Computed tomography (CT) revealed an irregularly enhanced tumor in the anterior mediastinum with a maximum size of ∼3 cm (Fig. 1A). Positron emission tomography/CT scans revealed increased ^18^F-fluorodeoxyglucose uptake in the mass (maximum standard uptake value, 3.35), although no abnormal uptake indicative of distant metastases was observed (Fig. 1B).

### Surgery

**Figure 2.**
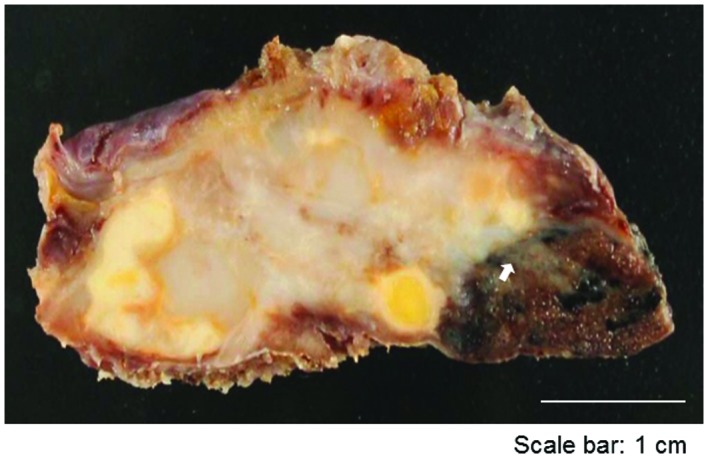
Macroscopic appearance of the cut sections of the tumor revealing a yellowish-white mass invading the left upper lobe (arrow).

Surgery was performed under the diagnosis of a suspected recurrent thymoma. A posterolateral thoracotomy was performed under video-assisted thoracoscopy. Severe adhesions were observed around the tumor, which appeared to have invaded the left upper lung and pericardium, while no pleural dissemination was observed. Therefore, the tumor was extirpated in combination with partial resection of the left upper lung and pericardium, and the excised pericardium was repaired using a polytetrafluoroethylene sheet. The tumor was found to be a yellowish-white solid mass invading the lung (Fig. 2).

### Histopathology

**Figure 3.**
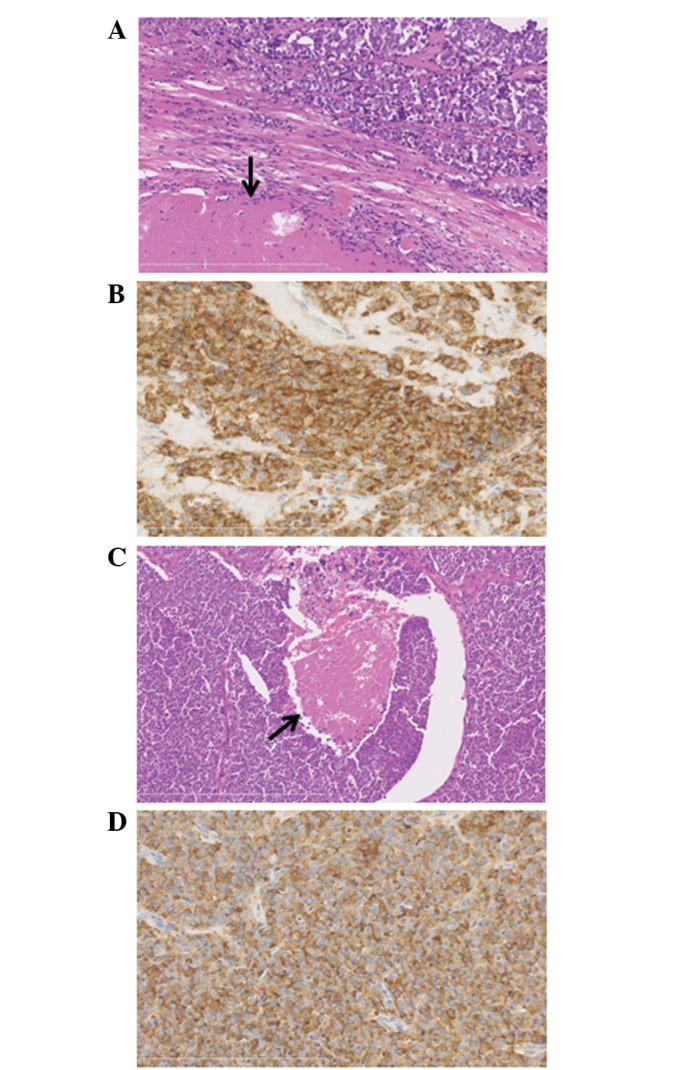
Microscopic features and immunohistochemical observations. (A) HE staining demonstrating that the tumor consisted of atypical carcinoid cells proliferating in sheets or small nested patterns accompanied by necrosis (arrow). (B) Immunoreactivity to chromogranin A. (C) HE staining and (D) immunohistochemistry of the specimen that was resected 24 years earlier demonstrating the same pathological observations of necrosis (arrow) and immunoreactivity to chromogranin A (original magnification, ×200). HE, hematoxylin and eosin.

Histopathologically, atypical carcinoid cells were observed to be arranged in sheets or small nested patterns accompanied by necrosis and lymphoid infiltration invading the surrounding adipose tissue and lungs, while extremely few mitotic cells were observed (Fig. 3A). An immunohistochemical analysis revealed that the tumor exhibited immunoreactivity to neuroendocrine markers, including chromogranin A (Fig. 3B). Based on these observations, the tumor was diagnosed as a well-differentiated NEC (atypical carcinoid, due to the presence of necrosis). The surgical margin of the lung was affected by the cancer cells. Retrospectively, the specimen that had been excised 24 years previously was re-examined and was reported to exhibit the same histology, HE results and immunoreactivity to the neuroendocrine markers as the present tumor (Figs. 3C and D).

There were no post-operative complications. Although the surgical margins were positive for cancer cells, no medical intervention was administered due to the patient’s age and the invasiveness of radiation and chemotherapy.

## Discussion

Thymic NEC is a rare type of neoplasm arising in the thymus, accounting for 2–4% of all anterior mediastinal tumors (1). This form of neoplasm has long been confused with thymoma, although Rosai and Higa described thymic NEC as a separate entity from thymoma in 1972 (7). Thymic NECs are predominantly or exclusively composed of neuroendocrine cells and must be distinguished from other typical thymic carcinomas with small numbers of neuroendocrine cells (8). Thymic NECs are divided into two groups, well- and poorly-differentiated, depending on the degree of tumor differentiation. The former group contains typical and atypical carcinoids classified according to the presence of necrosis and/or the number of mitotic cells, while the latter group includes large cell NEC and small cell carcinoma. This categorization is significant in that the prognosis of a well-differentiated NEC is improved compared with that of a poorly-differentiated NEC (8). In the present case, well-differentiated neuroendocrine cells were accompanied by necrotic components.

Local recurrence and distant metastasis develops frequently following surgical excision of thymic NECs (2–4). Wang *et al* previously reported that local recurrence or distant metastasis developed 15–60 months after surgery in 4/5 (80%) patients. In these cases, the sites of relapse included the chest wall, regional lymph nodes, bones and lungs (2). In addition, Fukai *et al* reported that distant metastases developed in 10/13 (76.9%) of patients who underwent total tumor resection, despite the absence of local recurrence (3). The study also reported intervals of 4–99 months between surgery and recurrence, comparable to that reported by Tiffet *et al* (22–83 months) (4). A study by Economopoulos *et al* identified recurrence in one case 9 years after surgery (5). However, to the best of our knowledge, there are no reports of any cases of recurrent thymic NEC relapsing 10–20 years after surgery. Therefore, the present case involves the longest period of time between the recurrence of thymic NEC and surgery. The optimal therapeutic modality for the treatment of recurrent disease has not been determined. However, due to the aggressive nature of tumors prone to recur or metastasize even following total excision, more aggressive treatments, including routine adjuvant chemotherapy and re-excision of recurrent tumors, as performed in the present case, may be required to reduce the incidence of local recurrence and distant metastasis, and therefore improve survival.

In conclusion, this study presents a case of a surgically-excised thymic NEC recurring >20 years after the initial excision. Thoracic oncologists must be aware that thymic NECs may recur ≥20 years after surgical treatment.
